# The exosome-like vesicles from osteoarthritic chondrocyte enhanced mature IL-1β production of macrophages and aggravated synovitis in osteoarthritis

**DOI:** 10.1038/s41419-019-1739-2

**Published:** 2019-07-08

**Authors:** Zhenhong Ni, Liang Kuang, Hangang Chen, Yangli Xie, Bin Zhang, Junjie Ouyang, Jiangyi Wu, Siru Zhou, Liang Chen, Nan Su, QiaoYan Tan, Xiaoqing Luo, Bo Chen, Shuai Chen, Liangjun Yin, Haiyang Huang, Xiaolan Du, Lin Chen

**Affiliations:** 1 Laboratory for the Rehabilitation of Traumatic Injuries, Laboratory of Trauma, Center of Bone Metabolism and Repair, State Key Laboratory of Trauma, Burns and Combined Injury, Research Institute of Surgery, Laboratory for Prevention and Rehabilitation of Military Training Related Injuries, Daping Hospital, Army Medical University (Third Military Medical University), 400042 Chongqing, China; 2 Center for Joint Surgery, Southwest Hospital, Army Medical University (Third Military Medical University), 400038 Chongqing, China; 3 Department of Spine Surgery, Institute of Surgery Research, Daping Hospital, Army Medical University (Third Military Medical University), 400042 Chongqing, China; 4 Department of Orthopedic Surgery, The Second Affiliated Hospital, Chongqing Medical University, 400010 Chongqing, China; 5 Department of Orthopedic Surgery, Qianjiang Nationality Hospital, 409000 Chongqing, China

**Keywords:** Osteoarthritis, Macroautophagy

## Abstract

Synovitis, a common clinical symptom for osteoarthritis (OA) patients, is highly related to OA pathological progression and pain manifestation. The activated synovial macrophages have been demonstrated to play an important role in synovitis, but the mechanisms about macrophage activation are still not clear. In this study, we found that the exosome-like vesicles from osteoarthritic chondrocytes could be a new biological factor to stimulate inflammasome activation and increase mature IL-1β production in macrophages. The degraded cartilage explants produced more exosome-like vesicles than the nondegraded ones, while the exosome-like vesicles from chondrocytes could enter into joint synovium tissue and macrophages. Moreover, the exosome-like vesicles from osteoarthritic chondrocytes enhanced the production of mature IL-1β in macrophages. These vesicles could inhibit ATG4B expression via miR-449a-5p, leading to inhibition of autophagy in LPS-primed macrophages. The decreased autophagy promoted the production of mitoROS, which further enhanced the inflammasome activation and subsequent IL-1β processing. Ultimately, the increase of mature IL-1β may aggravate synovial inflammation and promote the progression of OA disease. Our study provides a new perspective to understand the activation of synovial macrophages and synovitis in OA patients, which may be beneficial for therapeutic intervention in synovitis-related OA patients.

## Introduction

The synovium of osteoarthritis (OA) patients presents a typical characteristic of chronic and low-grade inflammation (hereafter refer to synovitis)^1,2^, which even precedes the radiographic detectable cartilage lesion^3^. The synovitis is highly related to OA clinical symptoms such as pain^4–9^ and pathological changes^10,11^. Moreover, the patients with synovitis have a higher risk of the subsequent development of cartilage loss^4,12–14^. In the multicenter osteoarthritis study, Felson et al. revealed that synovitis could be an independent cause of OA^15^. The occurrence of synovitis leads to the alternation of inflammatory cytokine profile in the joint, which could aggravate cartilage lesion and promote osteophyte formation^16,17^. In addition, some anti-inflammation strategies could efficaciously relieve OA pain, indicating that targeting joint inflammatory response could be a potential therapy in future^3^. However, the pathogenesis about synovitis is still not well clarified until now.

Previous studies reported that macrophages played a key role in the pathogenesis of synovitis^18^. Synovial macrophages and macrophage-produced mediators drive inflammatory and destructive responses in OA^2,19,20^. Taking the (99 m)Tc-EC20 (Etarfolatide) imaging technique, Kraus et al. found that the activated macrophages were present in the majority (76%) of OA knees, which was significantly associated with pain severity and radiographic OA severity^21^. Moreover, soluble macrophage biomarkers in synovial fluid appear to predict structural progression (CD163 and CD14) and pain (CD14) in OA knees^22^. In experimental animal OA model, activated macrophages are also abundantly detectable, especially in the early-stage^23^. The synovial macrophages are crucial in early MMP activity and appear to mediate MMP production in synovium rather than cartilage^24^. Depletion of synovial macrophages results in a significant reduction of osteophyte formation and OA-related pathology in experimental OA model^25^. Recently, Zhang et al. reported that M1 polarization of synovial macrophage remarkably exacerbates experimental OA, indicating that M1 macrophages are a potential therapeutic approach for OA treatment^26^. However, the mechanism of macrophage activation in synovium is poorly understood.

Exosome is one subtype of secreted vesicles, which could mediate communications between different cells and modulate multiple biological processes including immune response and inflammation^27–32^. Recently, the pathophysiological functions of exosomes in OA have also been explored. The exosomes from OA synovial fluid could increase the production of several M1-related cytokines in macrophages and presented obvious proinflammatory effect^33^, while Kolhe et al. also revealed that exosomes from OA synovial fluid significantly decreased the expression of anabolic genes and elevated the expressions of catabolic genes in chondrocytes^34^. In addition, the exosomes from IL-1β-stimulated synovial fibroblasts induce OA-like changes in vitro and in vivo^35^. These studies imply that exosomes may play an important role in OA progression. However, it is still unknown that whether the articular cartilage could produce exosome-like vesicles that mediate the communication between cartilage and synovium tissue.

In this study, we isolated and identified the exosome-like vesicles from chondrocytes. These exosome-like vesicles could enter into joint synovium tissue and mediate the communication between chondrocytes and macrophages. Moreover, the exosome-like vesicles from IL-1β-pretreated(osteoarthritic) chondrocytes significantly increased the production of mature IL-1β in macrophages. These vesicles could downregulate ATG4B expression via miR-449a-5p, leading to inhibition of autophagy in LPS-primed macrophages. The decreased autophagy can promote the production of mitochondrial ROS (mitoROS), which further enhanced the inflammasomes activation and mature IL-1β production. Ultimately, the increase of mature IL-1β may aggravate synovial inflammation and the damage of OA cartilage. Our study provides a new perspective to understand the activation of synovial macrophages and synovitis in OA patients.

## Results

### Separation and identification of chondrocytes-derived exosome-like vesicles

**Fig. 1:**
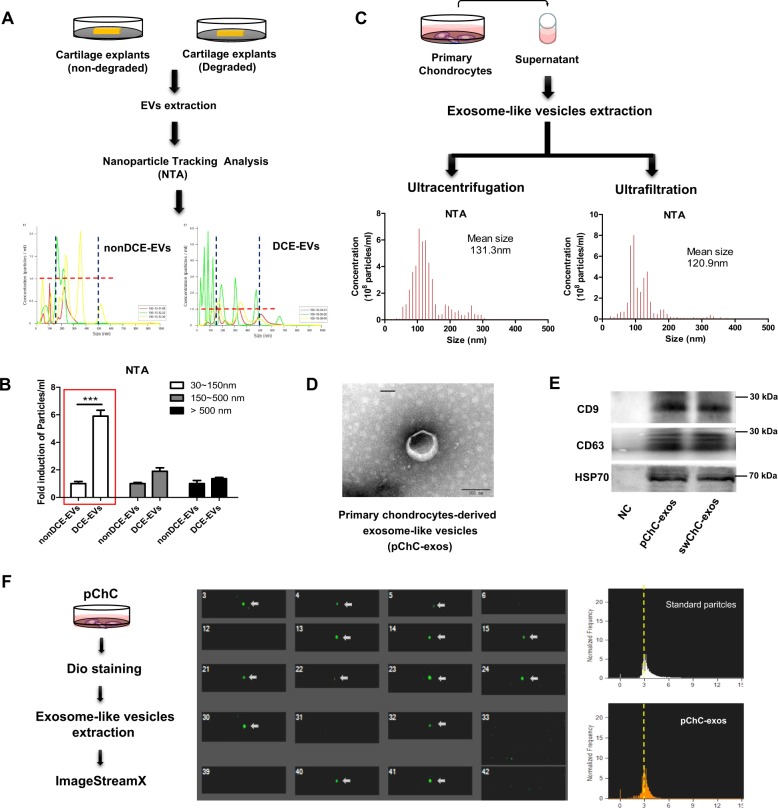
Separation and identification of chondrocytes-derived exosome-like vesicles. **a** The nondegraded cartilage explants or the degraded cartilage explants from donors were cultured in serum-free cell culture media. Then the supernatant extracellular vesicles obtained by ultrafiltration assay (10 KDa MWCO) were collected and subjected to NanoSight analysis. **b** The corresponding statistical graph for the size distribution of extracellular vesicles from different samples were shown (*n* = 3), ANOVA with Bonferroni’s multiple comparison test was used, ***p< 0.001. **c** Human primary chondrocytes from OA patients were isolated and cultured in serum-free cell culture media. Then the supernatant exosome-like vesicles extracted using ultracentrifugation assay (left) and ultrafiltration assay (right) were separately subjected to NanoSight detection. **d** The morphology of exosome-like vesicles from human primary chondrocytes were observed under transmission electron microscope, and the representative image was shown. **e** The indicated protein level in exosome-like vesicles from human primary chondrocytes were detected by Western blot. **f** The size distribution of Dio-labeled exosome-like vesicles from human primary chondrocytes were measured by ImageStreamX using 100 nm FITC-nanoparticles as standard control

Firstly, we obtained nondegraded and the degraded cartilage explants from OA donors and cultured these explants in serum-free cell culture media. Then the supernatant extracellular vesicles (EVs) were collected by conditional ultrafiltration assay (10 KDa MWCO) and further subjected to NanoSight detection (Fig. 1a). As shown in Fig. 1b, the number of EVs with the diameter among 30–150 nm from the degraded cartilage explants was dramatically increased (nearly 5.9-fold on average) compared with that from nondegraded cartilage explants, while the number of EVs with the diameter among the other ranges had no obvious change between theses cartilage explants. These data reveal that the degraded cartilage explants may produce or release more 30–150 nm extracellular vesicles (hereafter referred to as exosome-like vesicles) than nondegraded cartilage explants. Next, we obtained exosome-like vesicles from human primary chondrocytes using ultracentrifugation or ultrafiltration method. As shown in Fig. 1c, the diameters of the collected exosome-like vesicles mainly distributed among 30–150 nm, with the average particle size was 131.3 nm for ultracentrifugation-separated vesicles and 120.9 nm for ultrafiltration-separated vesicles. In addition, we observed the morphology of exosome-like vesicles derived from human primary chondrocytes using transmission electron microscope. The imaging showed that the exosome-like vesicles exhibited typical sphere-shaped bilayer membrane structure with the diameter of about 100 nm, which was the classical characteristics of exosomes (Fig. 1d). Moreover, we detected the expression of exosome-related markers in ultracentrifugation-separated exosome-like vesicles from human primary chondrocytes and SW1353 cells. As shown in Fig. 1e, the CD9, CD63, and HSP70 proteins were obviously detectable in these exosome-like vesicles. Additionally, the data from flow imaging assay also demonstrated that the diameters of most exosome-like vesicles from human primary chondrocytes were ~100 nm (Fig. 1f). Taken together, the above results indicate that chondrocytes could produce mounts of 30–150 nm exosome-like vesicles, which may dramatically increase when cartilage was degraded.

### The exosome-like vesicles from osteoarthritic chondrocyte promotes mature IL-1β production of macrophages

**Fig. 2:**
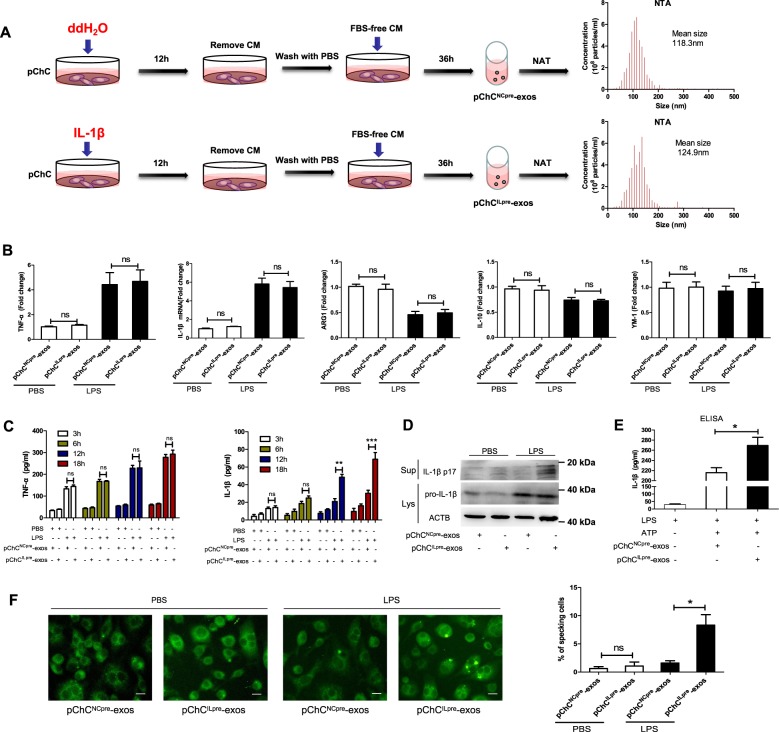
The exosome-like vesicles form osteoarthritic chondrocyte promote mature IL-1β production in macrophages. **a** Human primary chondrocytes were treated with 10 ng/ml of IL-1β or negative control ddH_2_O for 12 h and then washed with PBS twice. Subsequently, the chondrocytes were cultured in serum-free cell culture media for 36 h and the supernatant exosome-like vesicles were extracted using ultrafiltration assay for NanoSight analysis. pChC^ILpre^-exos, exosome-like vesicles from IL-1β-pretreated primary chondrocytes; pChC^NCpre^-exos, exosome-like vesicles from ddH_2_O-pretreated primary chondrocytes; **b** PMA-induced THP-1 cells were treated with pChC^NCpre^-exos or pChC^ILpre^-exos in the presence or absence of LPS and then the indicated mRNA level of treated cells was detected using qPCR assay. **c** PMA-induced THP-1 cells were treated with pChC^NCpre^-exos or pChC^ILpre^-exos in the presence or absence of LPS. Then the supernatant TNF-α and IL-1β were measured using ELISA assay. **d** The cells were treated as **c** and then the supernatant proteins were extracted for Western blot assay. **e** PMA-induced THP-1 cells were treated with pChC^NCpre^-exos or pChC^ILpre^-exos in the presence of LPS for 12 h, followed by 3 mM ATP for 30 min. Then the supernatant IL-1β were measured using ELISA assay. **f** PMA-induced THP-1 cells were treated with pChC^NCpre^-exos or pChC^ILpre^-exos in the presence or absence of LPS. Subsequently, the fluorescence-labeled ASC protein in the treated cells by immunofluorescence were observed under fluorescence microscope and the corresponding statistical graph for the ratio of ASC-specking cells were shown in the right. ANOVA with Bonferroni’s multiple comparison test was used, ***p < 0.001; **p < 0.01; *p < 0.05; ns, no significance

The EVs could be a way of communication between cartilage and synovial tissue^36^. Moreover, the exosomes could modulate the inflammatory signaling of macrophages including inflammasome activation^37–39^. Therefore, we further investigated the role of chondrocytes-derived exosome-like vesicles on macrophages. Firstly, we collected the exosome-like vesicles from human primary chondrocytes with pretreatment of ddH_2_O or IL-1β (a key pathogenic factor of OA), which were separately named as pChC^NCpre^-exos and pChC^ILpre^-exos (Fig. 2a). The data from Nanosight detection revealed that the concentration and size distribution of particles between pChC^NCpre^-exos and pChC^ILpre^-exos had no statistical differences (Fig. S1A, S1B). Then we used these two exosome-like vesicles to treat PMA-primed THP-1 cells with or without LPS and detected macrophage phenotype (M1/M2) using RT-PCR assay. As shown in Fig. 2b, pChC^ILpre^-exos did not significantly influence the mRNA level of M1/M2-related markers compared with pChC^NCpre^-exos. Next, we measured the supernatant levels of inflammatory cytokines (TNF-α and IL-1β) of THP-1 cells with pChC^NCpre^-exos or pChC^ILpre^-exos treatment in the presence or absence of LPS using ELISA assay. As shown in Fig. 2c, pChC^ILpre^-exos combined with LPS did not change the supernatant TNF-α level at 3 h, 6 h, 12 h, and 18 h, also the IL-1β level at 3 h and 6 h compared with pChC^NCpre^-exos. However, pChC^ILpre^-exos plus LPS obviously increased the supernatant IL-1β level at 12 h and 18 h compared with pChC^NCpre^-exos plus LPS (Fig. 2c). In addition, the data from western blot assay demonstrated that co-treatment of pChC^ILpre^-exos/LPS (not pChC^NCpre^-exos/LPS) significantly increased the level of supernatant mature IL-1β, while it had little effect on the cellular pro-IL-1β protein levels (Fig. 2d). Similar results were also shown in swChC^ILpre^-exos/LPS-treated THP-1 cells (Fig. S1C, S1D). These data indicate ChC^ILpre^-exos, but not ChC^NCpre^-exos, could promote IL-1β processing in LPS-primed macrophages. Moreover, pChC^ILpre^-exos also increased the production of supernatant IL-1β in the model of LPS/ATP-activated NLRP3 inflammasomes (Fig. 2e). In addition, the data from immunofluorescence assay revealed that the ratio of ASC-specking cells was increased in pChC^ILpre^-exos/LPS-treated cells (Fig. 2f), indicating an enhanced inflammasome activation in these cells. In brief, the above data revealed that the exosome-like vesicles from IL-1β-pretreated chondrocytes increased the production of mature IL-1β.

### Autophagy inhibition contributes to the ChC^ILpre^-exos-mediated IL-1β production

**Fig. 3:**
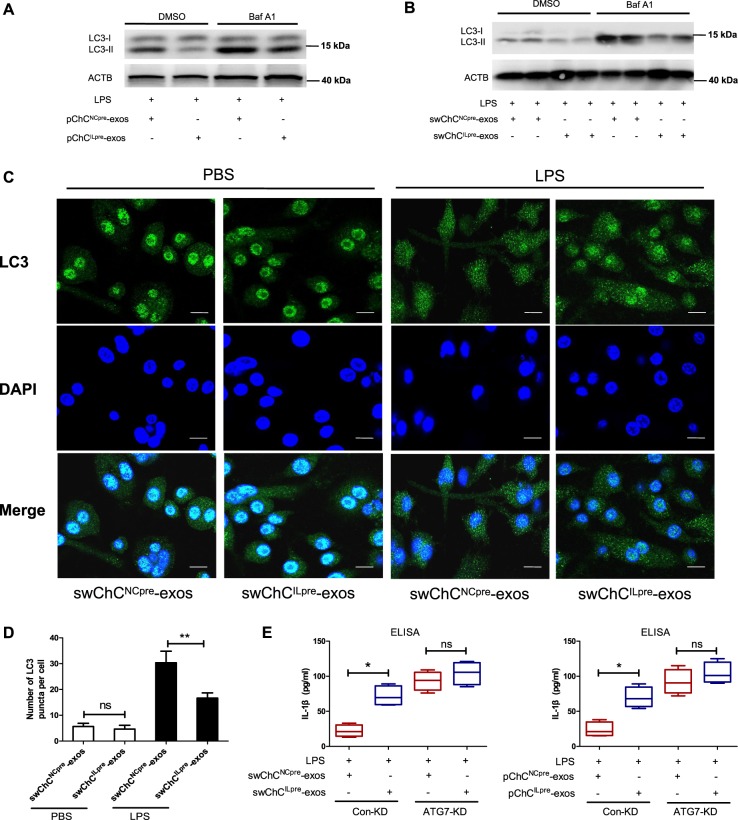
Autophagy inhibition contributes to ChC^ILpre^-exos-mediated production of mature IL-1β. **a** PMA-induced THP-1 cells were treated with pChC^NCpre^-exos or pChC^ILpre^-exos in the presence of LPS and then the indicated protein levels were detected by western blot assay. **b** PMA-induced THP-1 cells were treated with swChC^NCpre^-exos or swChC^ILpre^-exos in the presence of LPS for 24 h, combined with 100 nM Baf A1 for aftermost 3 h. Subsequently, the indicated protein levels were detected by Western blot assay. swChC^ILpre^-exos, exosome-like vesicles from IL-1β-pretreated SW1353 cells; swChC^NCpre^-exos, exosome-like vesicles from ddH_2_O-pretreated SW1353 cells. **c** PMA-induced THP-1 cells were given with the indicated treatments and then immunofluorescence was used to detect the expression pattern of LC3 (Green). DAPI staining was taken to mark the nucleus. The corresponding statistical graph for the average number of LC3 puncta per cell was shown (**d**). **e** PMA-induced THP-1 cells were transfected with ATG7 siRNA and the control siRNA and then given with the indicated treatments. Subsequently, the supernatant IL-1β was measured by ELISA assay. ANOVA with Bonferroni’s multiple comparison test was used, **p < 0.01; *p < 0.05; ns, no significance

It has been reported that autophagy could suppress inflammasome activation and decrease mature IL-1β production in macrophages^40^, so we deduced that whether ChC^ILpre^-exos-mediated production of mature IL-1β was related to autophagy inhibition. As shown in Fig. 3a, pChC^ILpre^-exos/LPS co-treatment in PMA-induced THP-1 cells significantly decreased the level of autophagy-related marker LC3-II compared with pChC^NCpre^-exos/LPS. In addition, the accumulation of LC3-II after the addition of BafA1 (autophagy inhibitor) was obviously decreased in pChC^ILpre^-exos/LPS-treated cells compared with that of pChC^NCpre^-exos/LPS-treated cells. Similar results were also observed in swChC^ILpre^-exos/LPS co-treated THP-1 cells (Fig. 3b) or mpChC^ILpre^-exos/LPS co-treated BMDMs (Fig. S2A). In addition, we also took immunofluorescence assay to detect the change of LC3-puncta, which could be considered as a marker for autophagosome. As shown in Fig. 3c, d, the LC3 signal distributed in the cytoplasm and nucleus in PMA-induced THP-1 cells. The LC3 signal in the nucleus was stronger than that in the cytoplasm when the cells were treated with swChC^NCpre^-exos or swChC^ILpre^-exos in the absence of LPS. After the addition of LPS, the LC3 signal in the nucleus was significantly decreased and the number of LC3-punta was obviously increased in swChC^NCpre^-exos- and swChC^ILpre^-exos-treated cells (Fig. 3c, d). Moreover, the number of LC3-punta in swChC^ILpre^-exos/LPS-treated cells was much less than that in swChC^NCpre^-exos/LPS-treated cells (Fig. 3c, d). The above data demonstrated that exosome-like vesicles from osteoarthritic chondrocytes dramatically inhibited the autophagy level in LPS-treated macrophages compared with the control vesicles. To evaluate the role of inhibited autophagy in ChC^ILpre^-exos-regulated mature IL-1β production, we further used siRNA to interfere autophagy-related gene ATG7 so as to inhibit autophagic flux in macrophages. The data from western blot showed an effective knockdown of ATG7 protein level in PMA-induced THP-1 cells with or without LPS treatment (Fig. S2B). The supernatant IL-1β was upregulated in ATG7-KD THP-1 cells with LPS treatment compared to that in the control cells (Fig. 3e), which was consistent with previous report^41^. Moreover, the statistic difference of the supernatant IL-1β level between ChC^NCpre^-exos/LPS-treated and ChC^ILpre^-exos/LPS-treated cells was eliminated when the ATG7 protein was knockdown (Fig. 3e). In brief, these data indicate that swChC^ILpre^-exos or pChC^ILpre^-exos-mediated inhibition on autophagy could contribute to the production of mature IL-1β in LPS-treated macrophages.

### ChC^ILpre^-exos-mediated production of mature IL-1β is partially dependent on the increased mitoROS

**Fig. 4:**
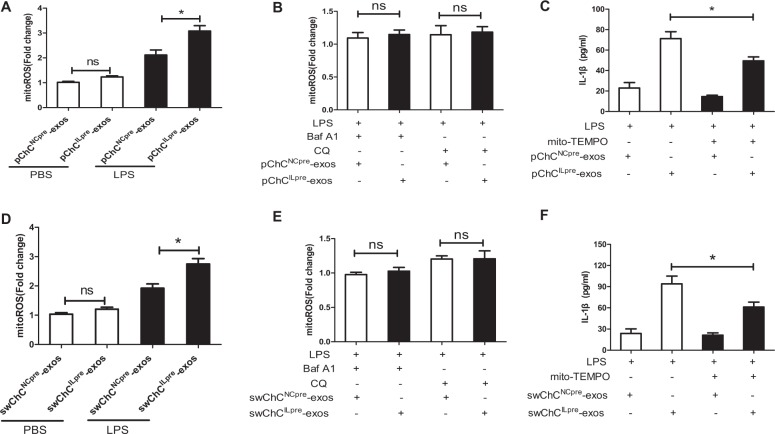
ChC^ILpre^-exos-mediated production of mature IL-1βis partially dependent on the increase of mitoROS. **a** PMA-induced THP-1 cells were treated with pChC^NCpre^-exos or pChC^ILpre^-exos in the presence or absence of LPS and then the level of mitochondrial ROS (mitoROS) were detected using MitoSox fluorescent probe. **b** PMA-induced THP-1 cells were treated with pChC^NCpre^-exos or pChC^ILpre^-exos in the presence of LPS for 24 h, combined with 100 nM Baf A1 or 20 μM CQ for aftermost 3 h. Then the level of mitoROS were measured. **c** PMA-induced THP-1 cells were pre-treated with 20 μM mito-TEMPO (a mitochondrial-specific ROS scavenger), followed with pChC^NCpre^-exos or pChC^ILpre^-exos in the presence of LPS for 24 h. Then the supernatant IL-1β were measured using ELISA assay. **d** PMA-induced THP-1 cells were treated with swChC^NCpre^-exos or swChC^ILpre^-exos in the presence or absence of LPS and then the level of mitoROS were detected. **e** PMA-induced THP-1 cells were treated with swChC^NCpre^-exos or swChC^ILpre^-exos in the presence of LPS for 24 h, combined with 100 nM Baf A1 or 20 μM CQ for aftermost 3 h. Then the level of mitoROS were measured. **f** PMA-induced THP-1 cells were pre-treated with 20 μM mito-TEMPO, followed with swChC^NCpre^-exos or swChC^ILpre^-exos in the presence of LPS for 24 h. Then the supernatant IL-1β was measured using ELISA assay. ANOVA with Bonferroni’s multiple comparison test was used, *p < 0.05; ns, no significance

Autophagy has been considered as an important mechanism to maintain the homeostasis of mitochondria including the level of reactive oxygen species (ROS)^42^. Moreover, ROS could be a potent signal to activate inflammasomes and promote mature IL-1β production^43^. Therefore, we further assessed the role of mitoROS in ChC^ILpre^-exos-mediated IL-1β production. Firstly, the change of mitoROS level was measured in pChC^NCpre^-exos- or pChC^ILpre^-exos-treated THP-1 cells with or without LPS. As shown in Fig. 4a, the cells with pChC^ILpre^-exos/LPS co-treatment presented higher level of mitoROS than that with pChC^NCpre^-exos/LPS co-treatment, while the mitoROS level did not show any difference between pChC^NCpre^-exos/PBS-treated and pChC^ILpre^-exos/PBS-treated cells. In addition, autophagy inhibitors Baf A1 or CQ eliminated the statistic difference of mitoROS between pChC^NCpre^-exos/LPS and pChC^ILpre^-exos/LPS group (Fig. 4b), indicating that pChC^ILpre^-exos-mediated up-regulation of mitoROS in LPS-treated cells was highly dependent on autophagy. Moreover, mitochondrial-specific ROS scavenger mitoTEMPO could partially reverse pChC^ILpre^-exos-mediated increase of mature IL-1β in LPS-treated cells (Fig. 4c), suggesting that mitoROS was involved in IL-1β processing in this model. Similarly, swChC^ILpre^-exos also induced higher mitoROS in LPS-treated cells than swChC^NCpre^-exos (Fig. 4d), while this difference was eliminated by the addition of CQ or Baf A1 (Fig. 4e). Moreover, swChC^ILpre^-exos-mediated increase of mature IL-1β was also partially reversed by mitoTEMPO in LPS-treated cells (Fig. 4f). Briefly, ChC^ILpre^-exos upregulated mitoROS level in LPS-treated macrophages via autophagy inhibition, which further enhanced the production of mature IL-1β.

### ATG4B plays an important role in ChC^ILpre^-exos-mediated inhibition of autophagy

**Fig. 5:**
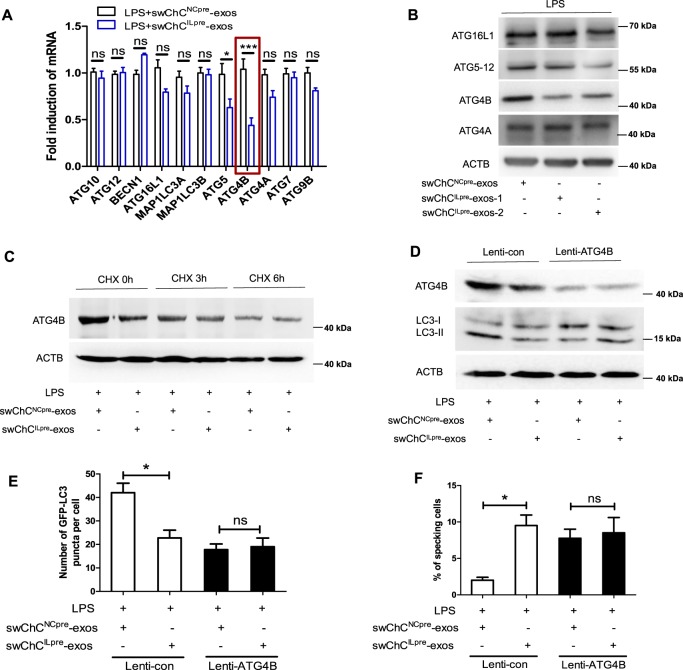
ATG4B plays an important role in ChC^ILpre^-exos-mediated inhibition of autophagy. **a** RT-PCR was used to detect the mRNA change of autophagy-related gene in PMA-induced THP-1 cells with the indicated treatments. **b** PMA-induced THP-1 cells were given with the indicated treatments for 12 h and then the ATG4B protein level was detected by western blot. **c** PMA-induced THP-1 cells were preincubated with 10 µM CHX for the indicated time, and then incubated swChC^NCpre^-exos or swChC^ILpre^-exos in the presence of LPS for an additional 12 h. Subsequently, the ATG4B protein level was evaluated by western blot. **d** PMA-induced THP-1 cells were transfected with Lenti-ATG4B shRNA or its control shRNA. Then the protein levels of ATG4B and LC3I/II were detected by western blot. **e**, **f** PMA-induced THP-1 cells were treated as **d** and then the immunofluorescence assay was taken to measure the LC3-puncta number per cell (**e**) and the ratio of ASC-specking cells (**f**). ANOVA with Bonferroni’s multiple comparison test was used, ***p < 0.001; *p < 0.05; ns, no significance

Next, we screened the changes of autophagy-related genes (Agts) using RT-PCR assay to investigate the potential mechanism of ChC^ILpre^-exos-mediated autophagy inhibition. As shown in Fig. 5a, the mRNA levels of several Agts including ATG16L1, ATG5, ATG4B, and ATG4A presented a decreased trend in swChC^ILpre^-exos/LPS co-treated cells, while the change of ATG4B mRNA was the most obvious among these genes. The data from western bolt assay also revealed that swChC^ILpre^-exos or pChC^ILpre^-exos significantly decreased the ATG4B protein level in LPS-treated cells (Figs. 5b and S3A). Cycloheximide (CHX), a protein synthesis inhibitor, eliminated swChC^ILpre^-exos-mediated decrease of ATG4B protein, indicating the decrease of ATG4B protein by swChC^ILpre^-exos strongly attributed to the inhibition of protein synthesis (Fig. 5c). Moreover, the level of LC3-II presented no difference between swChC^ILpre^-exos/LPS and swChC^NCpre^-exos/LPS groups when ATG4B was knockdown by lentivirus (Figs. 5d and S3B). The data from immunofluorescence assay showed that ATG4B inhibition abolished the difference of LC3-puncta or the ratio of ASC-specking cells between swChC^ILpre^-exos/LPS- and swChC^NCpre^-exos/LPS-treated cells (Fig. 5e, f). Taken together, the above results demonstrate that ChC^ILpre^-exos-mediated inhibition of autophagy and activation of inflammasomes was partially dependent on the decrease of ATG4B level.

### miR-449a-5p contributes to ChC^ILpre^-exos-mediated decrease of ATG4B level

**Fig. 6:**
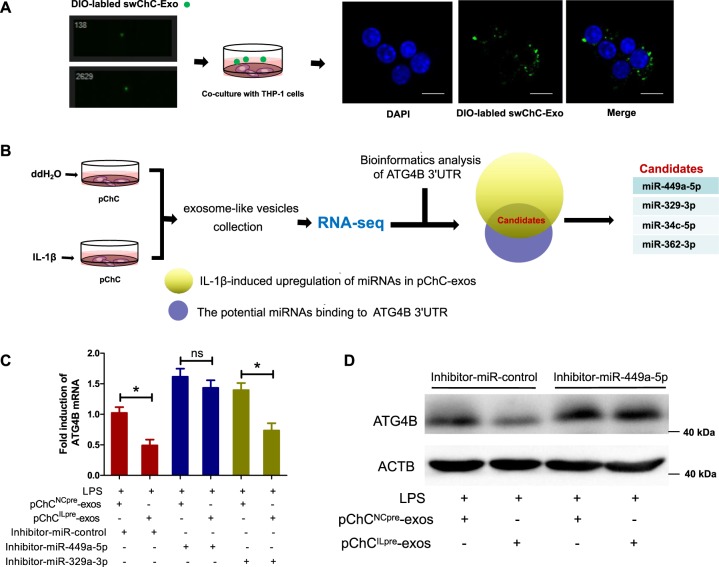
miR-449a-5p contributes to ChC^ILpre^-exos-mediated decrease of ATG4B level. **a** Dio-labeled swChC-exos were coincubated with PMA-induced THP-1 cells and then the distribution of green fluorescence was observed under the fluorescence microscope. **b** The total RNA from pChC^NCpre^-exos or pChC^ILpre^-exos were separately subjected to RNA sequencing analysis. The candidates from RNA-seq results and Bioinformatics analysis of ATG4B 3′UTR were listed. **c** PMA-induced THP-1 cells were transfected with the indicated miR-inhibitors, followed with pChC^NCpre^-exos or pChC^ILpre^-exos in the presence of LPS. Then the ATG4B mRNA were detected by RT-PCR. **d** PMA-induced THP-1 cells were transfected with the inhibitor-miR-449a-5p and the negative control, followed with pChC^NCpre^-exos or pChC^ILpre^-exos in the presence of LPS. Then the ATG4B protein level was detected by western blot. ANOVA with Bonferroni’s multiple comparison test was used, *p < 0.05; ns, no significance

Next, we labeled the SW1353-derived exosome-like vesicles (swChC-exos) using Dio-staining and detected whether these Dio-labeled swChC-exos can enter into macrophages. As shown in Fig. 6a, the green fluorescence signal of Dio-labeled swChC-exos was observed within the macrophages, especially in the cytoplasm of the cells, indicating that these exosome-like vesicles could enter into macrophages. Next, we took microRNA sequencing (miRNA-seq) to screen the potential differential miRNAs between pChC^ILpre^-exos and pChC^NCpre^-exos (Fig. 6b). Combined with the bioinformatic analysis of microRNA binding sites in ATG4B mRNA 3′UTR, we screened four candidates including miR-449a-5p, miR-329–3p, miR-34c-5p, and miR-362–3p. Among them, miR-449a-5p and miR-329–3p had the first and second highest level (Fig. 6b). To further verify the effect of miR-449a-5p and miR-329–3p on ChC^ILpre^-exos-mediated inhibition of ATG4B expression, we used microRNA inhibitors to decrease the level of miR-449a-5p and miR-329–3p in PMA-induced THP-1 cells. As shown in Fig. 6c, transfection of miR-449a-5p inhibitor, but not the corresponding control inhibitor or miR-329–3p inhibitor, effectively reversed pChC^ILpre^-exos-decreased ATG4B mRNA, suggesting miR-449a-5p played a key role in pChC^ILpre^-exos-regulated ATG4B mRNA. The comparative analysis of sequence conservation on ATG4B mRNA 3′UTR showed that the potential binding site of miR-449a-5p in ATG4B mRNA 3′UTR was relatively conserved among different species (Fig. S3C). Moreover, we detected the level of ATG4B protein in LPS-treated cells with pChC^NCpre^-exos or pChC^ILpre^-exos in the presence or absence of miR-449a-5p inhibitor transfection. As shown in Fig. 6d, transfection of miR-449a-5p inhibitor reversed pChC^ILpre^-exos-mediated decrease of ATG4B protein. In addition, we also found that IL-1β treatment significantly increased the level of miR-449a-5p in human primary chondrocytes (Fig. S3D), which is consistent with previous study, indicating that IL-1β stimuli would be an important factor for the enrichment of miR-449a-5p in chondrocytes-derived exosome-like vesicles. In brief, the above results reveal that ChC^ILpre^-exos inhibits ATG4B expression via targeting miR-449a-5p in macrophages.

### The miR-449a-5p inhibitor partially reverse ChC^ILpre^-exos-mediated production of mature IL-1β

**Fig. 7:**
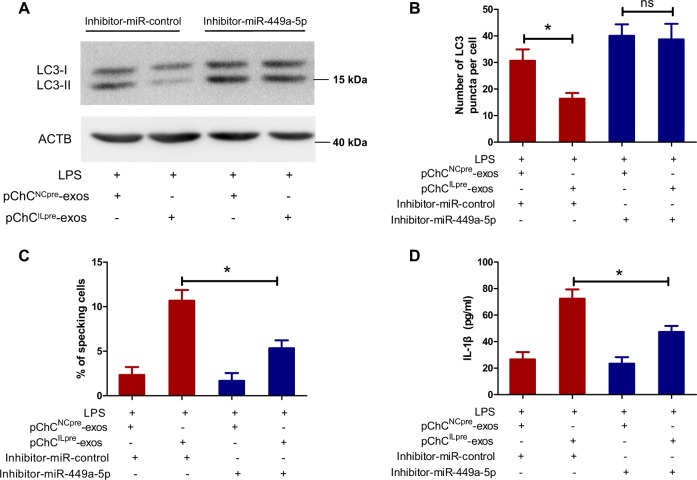
The miR-449a-5p inhibitor partially reverse the ChC^ILpre^-exos-mediated autophagy inhibition and production of mature IL-1β. **a** PMA-induced THP-1 cells were transfected with the inhibitor-miR-449a-5p and the negative control, followed with pChC^NCpre^-exos or pChC^ILpre^-exos in the presence of LPS. Then the LC3I/II protein level was detected by Western blot. **b**, **c** PMA-induced THP-1 cells were treated as **a** and then the immunofluorescence assay was taken to measure the LC3-puncta number per cell (**b**) and the ratio of ASC-specking cells (**c**). **d** PMA-induced THP-1 cells were treated as **a** and then the supernatant IL-1β was measured using ELISA assay. ANOVA with Bonferroni’s multiple comparison test was used, *p < 0.05; ns, no significance

As miR-449a-5p plays a key role in ChC^ILpre^-exos-mediated inhibition of ATG4B expression, we further evaluated the effect of miR-449a-5p on autophagy in this model. As shown in Fig. 7a, transfection of miR-449a-5p inhibitor abolished ChC^ILpre^-exos-mediated inhibition of LC3-II level (Fig. 7a). In addition, the data from immunofluorescence assay also revealed that the number of LC3-puncta has no statistical difference between LPS/ChC^ILpre^-exos and LPS/ChC^NCpre^-exos treated cells in the presence of miR-449a-5p inhibitor (Fig. 7b). Moreover, the ratio of ASC-specking cells and the supernatant IL-1β level were both significantly decreased in miR-449a-5p inhibitor-transfected cells compared with that in control inhibitor-transfected cells (Fig. 7c, d). Briefly, the above results demonstrate that ChC^ILpre^-exos-mediated production of mature IL-1β was highly related to miR-449a-5p.

### Intra-articular injection of ChC^ILpre^-exos aggravates cartilage erosion and synovitis in DMM-induced OA mice

**Fig. 8:**
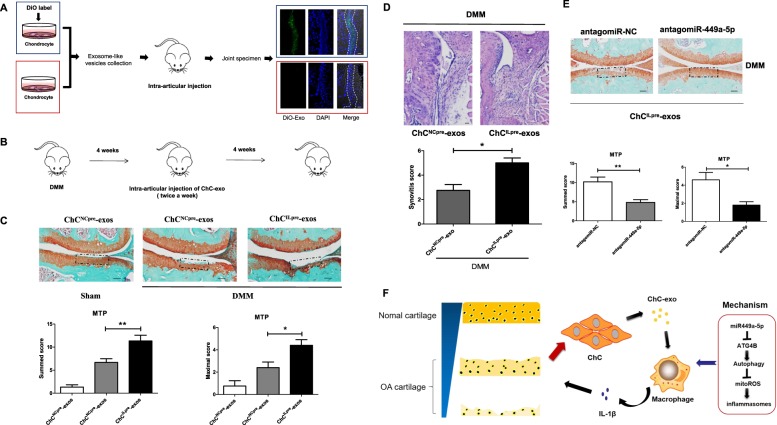
Intra-articular injection of ChC^ILpre^-exos aggravates cartilage erosion and synovitis in DMM-induced OA mice. **a** The human primary chondrocytes were labeled with or without Dio fluorescent dye. Then the supernatant exosome-like vesicles were separately extracted and injected to articular cavity of DMM-induced OA mouse. Then the Dio fluorescent signal in joint synovium was observed under the fluorescence microscope. **b** The DMM-induced OA mice were given with ChC^NCpre^-exos or ChC^ILpre^-exos via intra-articular injection as the indicated pattern in schematic diagram. **c** After 8 weeks of DMM operation, the joint samples were obtained for safranine O/fast green staining, taking the samples from sham group with intra-articular injection of ChC^NCpre^-exos as controls. The representative images and the corresponding statistical graphs were shown. ANOVA with Bonferroni’s multiple comparison test was used, **p < 0.01; *p < 0.05. **d** The DMM-induced OA mice were given with ChC^NCpre^-exos or ChC^ILpre^-exos via intra-articular injection as (**b**). After 6 weeks of DMM operation, the joint samples were obtained for hematoxylin-eosin staining and the synovitis scoring. Student’s *t* test, *p < 0.05. **e** The DMM-induced OA mice were given with intra-articular injection of antagomiR-NC (negative control) or antagomiR-449a-5p twice a week from the 4th week, combined with ChC^ILpre^-exos injection. After 8 weeks of DMM operation, the joint samples were obtained for safranine O/fast green staining. The representative images and the corresponding statistical graphs were shown. Student’s *t* test, **p < 0.01; *p < 0.05. **f** Chondrocytes could produce mounts of exosome-like vesicles and released from cartilage accompanied with cartilage matrix loss and cartilage erosion. These exosome-like vesicles could be highly enriched in synovial tissue and can enter into macrophages. The ChC^ILpre^-exos could deliver miR-449a-5p into macrophages and inhibit ATG4B expression in LPS-primed macrophages, which leads to inhibition of autophagy. The decreased autophagy promotes the production of mitoROS, which further enhances the inflammasome activation and mature IL-1β production. Ultimately, the increase of IL-1β aggravates synovial inflammation and promotes the progress of OA disease. ChC, chondrocytes; exo, exosome-like vesicles

Next, we evaluated the effect of ChC^ILpre^-exos on joint synovial inflammation and cartilage erosion in vivo. Firstly, we labeled the exosome-like vesicles from primary chondrocytes using Dio fluorescent dye and injected these vesicles to the articular cavity of destabilization of the medial meniscus (DMM)-induced OA mice to observe their distribution in joint. As shown in Fig. 8a, the Dio-labeled exosome-like vesicles could be highly detected in the synovial tissue of OA joint, indicating that chondrocytes-derived vesicles could be a mode of communication between cartilage and synovium. Subsequently, intra-articular injection of ChC^ILpre^-exos or ChC^NCpre^-exos was performed in DMM-induced OA mice and the cartilage damage was observed by Safranin O/Fast Green staining (Fig. 8b). The data from Fig. 8c showed that the destruction of articular cartilage in DMM mice with ChC^ILpre^-exos treatment was more severe than that with ChC^NCpre^-exos treatment. The Osteoarthritis Research Society International (OARSI) scoring including the summed scores and maximal scores of the tibia also revealed that intra-articular injection of ChC^ILpre^-exos aggravated the cartilage erosion in experimental OA model compared to ChC^NCpre^-exos (Fig. 8c). The synovial inflammation in ChC^ILpre^-exos-treated mice was more severe compared to ChC^NCpre^-exos-treated mice, such as synovial lining hyperplasia and infiltration of inflammatory cells (Fig. 8d). Next, intra-articular injection of antagomiR-NC or antagomiR-449a-5p (antagomiR-449a-5p could be used to inhibit the function of miR-449a-5p in vivo) in ChC^ILpre^-exos-treated DMM mice was performed to estimate the effect of miR-449a-5p on ChC^ILpre^-exos-mediated cartilage erosion. As shown in Fig. 8e, the articular cartilage destruction was more severe in mice with antagomiR-NC treatment than that with antagomiR-449a-5p treatment. In brief, the above results demonstrate that intra-articular injection of ChC^ILpre^-exos aggravated cartilage erosion and synovitis in DMM-induced OA mice, while antagomiR-449a-5p could partially reverse ChC^ILpre^-exos-mediated cartilage damage.

## Discussion

Taken together, the data of this manuscript suggest that OA-derived exosome-like vesicles could enriched in joint synovium and enter into macrophages. The exosome-like vesicles from osteoarthritic chondrocytes could increase the production of mature IL-1β partially via miR-449a-5p/ATG4B-mediated autophagy inhibition, which may further aggravate the synovitis and cartilage erosion in OA (Fig. 8f).

Cell-derived EVsEVs including exosomes, microvesicles, and apoptotic bodies in synovial fluid and cartilage extracellular matrix (ECM) are highly related to the regulation of joint homeostasis^44^. Headland et al. reported that neutrophil-derived microvesicles enter cartilage and protect the joint in inflammatory arthritis^36^. In this study, we found that Dio-labeled exosome-like vesicles could enter into synovium tissue, indicating the crosstalk between synovium and cartilage tissue using EVs. Intra-articular injection of exosomes can effectively promote cartilage tissue regeneration and prevent OA progression^45–49^. Moreover, Mitton et al. reported that articular cartilage vesicles (ACVs), 50–150 nm membrane-bound extracellular organelles, also contain RNA and these ACVs specifically transfer their labeled RNA and protein to intact primary chondrocytes^50^. Though the ACV proteome shares fewer similarities to exosomal proteomes^51^, these vesicles could be released from the matrix and interact directly with chondrocytes to promote chondrocyte hypertrophy during early OA. Moreover, RNA is selectively packaged into matrix vesicles (MVs) secreted by chondrocytes and well-protected from degradation by the lipid membrane, which are related to bone formation and metabolic signaling pathways^52^. More researches were needed to investigate the difference of MVs and exosome-like vesicles from cartilage.

The exosomes could have proinflammatory effect in different models. The circulating exosomes could effectively reach the alveolar compartment and were internalized by macrophages, while the exosomes obtained under inflammatory conditions activate and polarize the alveolar macrophages toward a proinflammatory phenotype^53^. The exosomes derived from alcohol-treated hepatocytes sensitize monocytes to LPS via transferring liver specific miRNA-122^54^. Mesenteric lymph-derived exosomes stimulated NF-κB activation and caused proinflammatory cytokine production in alveolar macrophages of rats after trauma/hemorrhagic shock^55^. Recently, Pan et al. found that adipocyte-secreted exosomes could inhibits M2 macrophage polarization to promote obesity-induced adipose inflammation via delivering microRNA-34a into the recipient cells^56^. Moreover, Zhang et al. reported that inflammasome-derived exosomes significantly activate the NF-κB signaling pathway in macrophages, suggesting that the inflammatory signaling could be amplified in neighbor cells in an exosome-dependent way^39^. Consistent with these studies, our data showed that exosome-like vesicles from IL-1β-pretreated chondrocytes obviously stimulated inflammasome activation in macrophages compared to the ones from ddH_2_O-pretreated chondrocytes, indicating that the exosomes from osteoarthritic chondrocytes (IL-1β-treated chondrocytes) may be more capable to activate inflammatory response of synovial macrophages. Of course, more studies are needed to investigate the role of these vesicles on other recipient cells in the joint.

The microRNA-449a has been demonstrated to possess different biological functions including the regulation of OA progression. Park et al. reported that the level of microRNA-449a was increased in OA patients-derived chondrocytes and IL-1β-treated chondrocytes^57^. Similarly, our results also revealed that IL-1β treatment significantly increase the expression of microRNA-449a-5p (Fig. S3D). Moreover, Park et al. found that suppression of microRNA-449a attenuated the expression of catabolic genes induced by IL-1β^57^. In addition, Baek et al. reported that inhibition of miR-449a could dramatically promote cartilage regeneration and prevent OA progression in rat models^58^. Wu et al. also found that the level of miR-449a was upregulated in OA cartilage compared to that in normal cartilage and overexpression of miR-449a contributed to ECM degradation of chondrocytes in OA via directly targeting GDF5^59^. The above studies suggest that microRNA-449a was increased in OA chondrocytes (especially under IL-1β stimulation) and may aggravate OA progression. In this study, our data indicated that inhibition of miR-449a-5p could alleviate OA-like pathologic changes in ChC^ILpre^-exos-treated DMM mice, suggesting the miR-449a-5p may impact the OA procession in this model. Nevertheless, more researches using genetically modified mice of miR-449a-5p were still needed to elucidate the role of miR-449a-5p on OA procession in vivo. In addition, our present data in Fig. 6c, d only demonstrated that miR-449a-5p contributes to ChC^ILpre^-exos-mediated decrease of ATG4B mRNA and protein level, but the direct target confirmation between miR-449a-5p and ATG4B in macrophages will still need further investigation.

## Materials and methods

### Isolation and culture of primary chondrocytes

Human cartilage samples were obtained from OA patients with the operation of total knee replacement in southwest hospital (Chongqing, China). The collected cartilage specimens were washed using sterile PBS that contained penicillin-streptomycin and diced by the bistoury. Subsequently, the diced cartilage was digested overnight in high-glucose DMEM supplemented with 0.2% type II collagenase. Then the cell suspension was filtered by a 40 μm cell strainer and the collected cells were subjected to centrifugation at 400 g for 5 min. At last, the pellets (primary chondrocytes) were resuspended in 10% FBS-contained high glucose DMEM and cultured in 5% C0_2_ incubator.

### Extract of exosome-like vesicles

#### ultracentrifugation for the extraction of exosome-like vesicles

The ultracentrifugation for the extraction of exosome-like vesicles in this study was performed according to the processes described previously^60^. Briefly, the culture supernatant was subjected to centrifugation at 300 g for 10 min and the pellet was removed. Then the supernatant was continued to centrifugation at 2000 g for 10 min and the pellet was removed. Subsequently, the supernatant was subjected to centrifugation at 10,000 g for 30 min and 100,000 g for 70 min. After that, the pellet was collected and washed with PBS. At last, the pellet-contained PBS was subjected to centrifugation at 100,000 g for 70 min and the pellet was collected for Nanosight detection and western blot assay.

#### ultrafiltration for the extraction of exosome-like vesicles

The culture supernatant was subjected to centrifugation at 300 g for 10 min and the pellet was removed. Then the supernatant was continued to centrifugation at 3000 g for 30 min and the pellet was removed. Then, the supernatant was filtered with a 0.22-μm filter (Steritop; Millipore) using a 5 ml needle. Subsequently, the 14 ml supernatant was added into the 10 kDa MWCO ultra-clear centrifuge tube (Millipore) and centrifuged at 4300 g for 40 min, and condensed fluid in the ultra-clear centrifuge tube was preserved. Next, the condensed fluid was diluted by another 14 ml culture supernatant and centrifuged at 4300 g for 40 min. After concentrating all the supernatant, the condensed fluid (nearly 200 μl) was washed with sterile PBS and subjected to centrifugation at 4300 g for 40 min. Finally, the condensed PBS that contained pellet (exosome-like vesicles) was obtained and used for the later experiments.

### Nanosight

The collected exosome-like vesicles by ultracentrifugation or ultrafiltration were adequately resuspended in 1 ml PBS and slowly loaded into the sample pool of Nanosight LM 10 (Malvern) using a syringe. Then the laser module was put back on the base and the probe of thermometer was put into the copper hole of laser module panel. Subsequently, the measurement parameters of Nanosight LM 10 were accordingly adjusted. After the sample measurement was completed, the pipeline was cleaned, and the data were further analyzed. The particle concentration (particles/ml) was calculated.

### Isolation and culture of primary mouse BMDMs

The tibia and femur of hind legs were detached from C57BL/6 J mice and washed with sterile PBS contained penicillin-streptomycin. Then the bone marrow was flushed with 10% FBS-contained RPMI-1640 using 5 ml syringe and the cell suspension was processed by nylon filter mesh. Subsequently, the filtered suspension was centrifuged at 1500 rpm for 5 min and the supernatant was removed. Next, the precipitate was resuspended by red blood cell lysis buffer at 4 °C for 10 min. At last, the cells without red blood cell were collected and cultured in 10 cm dish for 5 days in the presence of M-CSF (50 ng/ml).

### Western blot

The western blot assay was performed according to the method described previously^61^. In brief, the total protein was loaded to SDS-PAGE gel for electrophoresis and then transferred to PVDF (polyvinylidene fluoride) membrane. Subsequently, the PVDF membrane was orderly incubated with primary antibodies and secondary antibodies. At last, the enhanced chemiluminescence was added to react with secondary antibodies and the images were obtained. The antibodies used were CD9-antibody (60232–1-Ig, Proteintech), CD63-antibody (25682–1-AP, Proteintech), HSP70-antibody (10995–1-AP, Proteintech), IL-1β-antibody (#12242, Cell signaling), Cleaved-IL-1β-antibody (#83186, Cell signaling), ACTB/β-actin-antibody (A8481, Sigma-Aldrich), LC3B-antibody (#3868,Cell signaling), ATG4B-antibody (15131–1-AP, Proteintech), ATG5-antibody (10181–2-AP, Proteintech), ATG4A-antibody (27467–1-AP Proteintech), and ATG16L1-antibody (19812–1-AP, Proteintech).

### Flow imaging

Firstly, the exosome-like vesicles were stained with lipophilic green fluorescent dye (DiO) according to the manufacturer’s instruction. In brief, the cells with the concentration of 1 × 10^6^/ml were incubated with 5 μl/ml Dio fluorescent dye for 20 min at 37 °C and then the cells were washed with sterile PBS. Subsequently, the cells were continued to culture for 24 h and the supernatant was subject to the isolation of exosome-like vesicles (Dio-labeled exosome-like vesicles). Secondly, the size distribution of Dio-labeled exosome-like vesicles were measured by amnis ImageStream, taking the 100-nm FITC particles as the standard control.

### RT-PCR

Trizol reagent (ambion, invitrogen) was taken to extract the total RNA and then the first-strand cDNA was synthesized using PrimeScript^TM^ RT reagent kit with gDNA eraser according to the manufacturer’s instruction. RT-PCR was performed using SYBR select master mix in a 20 l volume. The primer sets for qPCR were listed below.

**Table:** 

Gene	Forward primer	Reverse primer
ATG10	AACGTCTCAGGATGAACGAAATG	TCGTGCCGCAATTCCTAAAC
ATG12	CTGCTGGCGACACCAAGAAA	CGTGTTCGCTCTACTGCCC
ATG16L1	ATTCAGTGCACCTGGGTTCAA	GTAACTGCCATCAGGGCTGAAG
ATG4A	CAACGCATCCTACAGTGCTTCTT	TACACCCATTTGTGCCATTTGA
ATG4B	ATTGGTGCCAGCAAGTCAA	GCAGGCCAGATGTGAAGG
ATG5	AAAACCCATTCCTTCCAAGCTAGT	CTGCCAGGGACCACAGTGA
ATG7	GGATGGCCTTTGAGGAATTTTT	GGTCACGGAAGCAAACAACTTC
ATG9B	GATGCGTGGATTACAATGTTCTCTT	GAGGGTAGGATGGCATCTGACA
MAP1LC3A	CTTCGCCGACCGCTGTAA	CCTTGTAGCGCTCGATGATCA
MAP1LC3B	CCCTGGAGAAAGAGTGGCATT	CTTTCCGTAACAACACAGGCACTA
BECN1	GAGCTGGAAGACGTGGAAAAGA	AGCCTGGACCTTCTCGAGATTT

### ELISA

The level of supernatant IL-1β was detected by mouse IL-1β ELISA Kit (Beyotime Biotechnology, PI301) or human IL-1βELISA Kit (Beyotime Biotechnology, PI305) in triplicate according to the manufacturer’s instruction. The OD450 value was recorded using microplate reader (multiscan spectrum, Thermo).

### Immunohistochemical assay and histological analysis

The immunohistochemical assay was performed as previously described^62^. In brief, the 5μm sections were deparaffinized with xylene and rehydrated. Subsequently, 3% H_2_O_2_ was added to block endogenous peroxidase activity. Then the sections were treated with 0.1% trypsin for antigen retrieval and normal goat serum for blocking. After that, the sections were orderly incubated with the primary antibodies at 4 °C overnight and secondary antibody at 37 °C for 1 h. At last, the horseradish peroxidase-conjugated streptavidin-biotin was added and the immunoreactivity was visualized under an optical microscope. The histopathological grading for cartilage destruction of knee joint was performed according to the recommendations of the OARSI^63^, while the histopathological grading for joint synovitis was carried out based on the methods previously described^64^.

### The extract of supernatant protein

The culture medium (0.5 ml) was added with the isometric methanol and fully mixed. Then 0.125 ml chloroform was added and the mixed solution was centrifuged at 13000 rpm for 10 min (4 °C). Subsequently, the liquid at the upper layer was slightly removed, the remainder liquid was added with 0.5 ml methanol and centrifuged at 15,000 rpm for 5 min (4 °C). At last, the supernatant was removed and the precipitate was added with 150 µl loading buffer-contained lysate for the follow experiments.

### mitoROS detection

The treated cells were washed with sterile PBS and then incubated with 3 μM MitoSOX-Red (Life Technologies, USA) in serum-free culture medium for 20 min at 37 °C. Subsequently, the stained cells were collected for FACScalibur flow cytometry detection and the data from three independent samples were calculated.

### exosomal miRNA-seq

The pChC^ILpre^-exos and pChC^NCpre^-exos were obtained from 60 ml cell culture supernatant and subjected to exosomal miRNA-seq by Guangzhou RiboBio Co., LTD.

### Experimental OA mice model

The right knee joints of 8-weeks-old male mice were subjected to DMM surgery according to the method previously described^65^, taking the left knee joints with medial capsulotomy as negative control. Four weeks after operation, mice were intra-articularly injected with exosome-like vesicles (10^9^ paritcals in 5 µl vehicle per knee joint) or exosome-like vesicles combined with antagomiR (5 nM in 5 µl vehicle per knee joint) biweekly during the following 4 weeks. After 8 weeks of operation, the mice were killed and the joint samples were subjected to pathological analysis.

### Transfection of microRNA inhibitors and lenti-shATG4B

Inhibitor-miR-control, inhibitor-miR-449a-5p, and inhibitor-miR-329a-3p were purchased from RiboBio Co., LTD (Guangzhou, China). The transfection of microRNA inhibitors in PMA-induced THP-1 cells was performed using riboFECT^TM^CP reagent (C10511–05) according to the manufacturer’s instruction. The pLVX-shRNA-ATG4B plasmid was constructed and packaged with lentiviral vector (lenti-shATG4B) by GeneCopoeia Co.,LTD (Guangzhou, China). The targeting sequence of lenti-shATG4B was 5′-GGTGTGGACAGATGATCTT-3′ and the MOI of transfection in THP-1 cells was 60.

### Statistics

All the data were analyzed using GraphPad Prism software. The results were presented as mean ± SD from three independent experiments. Differences between two groups were analyzed using Unpaired Student’s *t* test. Analysis of variance (ANOVA) with Bonferroni’s multiple comparison test was used for the comparisons of data in more than two groups of variables. *p < 0.05, **p < 0.01, and ***p < 0.001 were considered statistically significant.

## Supplementary information

Supplementary Figure legends

Supplementary Figures
