# Supplementary material for: The exosome-like vesicles from osteoarthritic chondrocyte enhanced mature IL-1β production of macrophages and aggravated synovitis in osteoarthritis
Source: Cell Death Dis. 2019 Jul 8;10(7):522. doi: 10.1038/s41419-019-1739-2 (PMC6614358; doi:10.1038/s41419-019-1739-2)
Supplement: Supplementary file 1 — Supplementary Figure legends [file 41419_2019_1739_MOESM1_ESM.pdf]

Figure S1 The exosome-like vesicles from IL-1β-pretreated chondrocytes promotes NLRP3 inflammasome activation of macrophages.

(A and B) Human primary chondrocytes were treated with 10 ng/ml of IL-1β or negative control ddH_2O for 12h and then washed with PBS twice. Subsequently, the chondrocytes were cultured in serum-free cell culture media for 36h and the supernatant exosome-like vesicles were extracted using ultrafiltration assay for NanoSight analysis. The corresponding statistical graph for the concentration of supernatant particals and the mean size of the particals were separately shown. Student's t test, ns, no significance. (C) PMA-induced THP-1 cells were treated with swChC^{NCpre}-exos or swChC^{ILpre}-exos in the presence or absence of LPS. Then the supernatant proteins were extracted for western blot assay. (D) PMA-induced THP-1 cells were treated as (B) and then the supernatant IL-1β were measured using ELISA assay. ANOVA with Bonferroni's multiple comparison test was used, *P < 0.05; ns, no significance.

Figure S2 ChC^{ILpre}-exos inhibited autophagy level of macrophages.

(A) Mouse primary chondrocytes were treated with 10 ng/ml of IL-1β or negative control ddH_2O for 12h and then washed with PBS twice. Subsequently, the chondrocytes were cultured in serum-free cell culture media for 36h and the supernatant exosome-like vesicles were extracted using ultrafiltration assay. Then the exosome-like vesicles combined with LPS were taken to treat mouse bone marrow-derived macrophages (BMDMs) for 12h, in the presence or absence of 100 nM Baf A1 for aftermost 3h. Then the total proteins were extracted for western blot assay. (B) PMA-induced THP-1 cells were transfected with ATG7 siRNA and the control siRNA and then treated with PBS or LPS for 12h. Subsequently, the ATG7 protein level was detected using western blot assay.

Figure S3 ChC^{ILpre}-exos decreased ATG4B level of macrophages.

(A) PMA-induced THP-1 cells were treated with pChC^{NCpre}-exos or pChC^{ILpre}-exos and then the total proteins were extracted for western blot assay. (B)The statistical analysis of ATG4B and LC3-II protein level in western blot assay. (C) Bioinformatics analysis for the potential binding site of ATG4B 3'UTR and miR-449a-5p, and the conservation of miR-449a-5p among different species. (D) Human primary chondrocytes were treated with 10 ng/ml of IL-1β for 6h and then the miR-449a-5p level were detected with by RT-PCR. ANOVA with Bonferroni's multiple comparison test was used, ***P < 0.001; *P < 0.05; ns, no significance.
