# Supplementary figures and images for: The exosome-like vesicles from osteoarthritic chondrocyte enhanced mature IL-1β production of macrophages and aggravated synovitis in osteoarthritis
Source: Cell Death Dis. 2019 Jul 8;10(7):522. doi: 10.1038/s41419-019-1739-2 (PMC6614358; doi:10.1038/s41419-019-1739-2)

# Figure S1

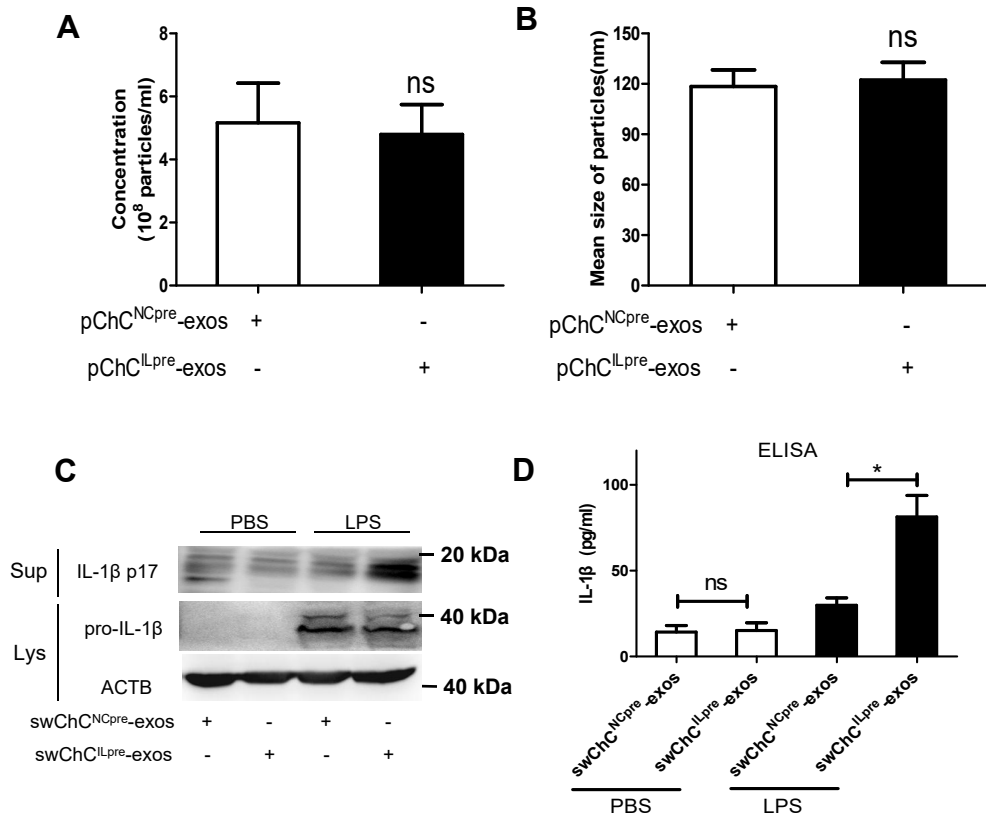

Figure S2

**A**

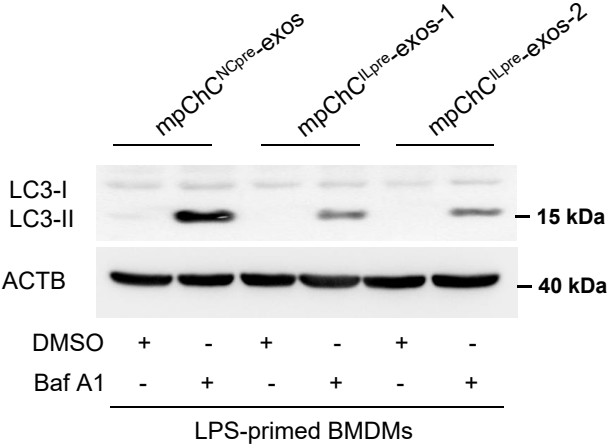

**B**

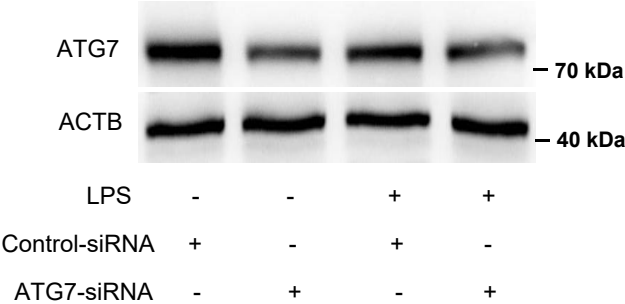

Figure S3

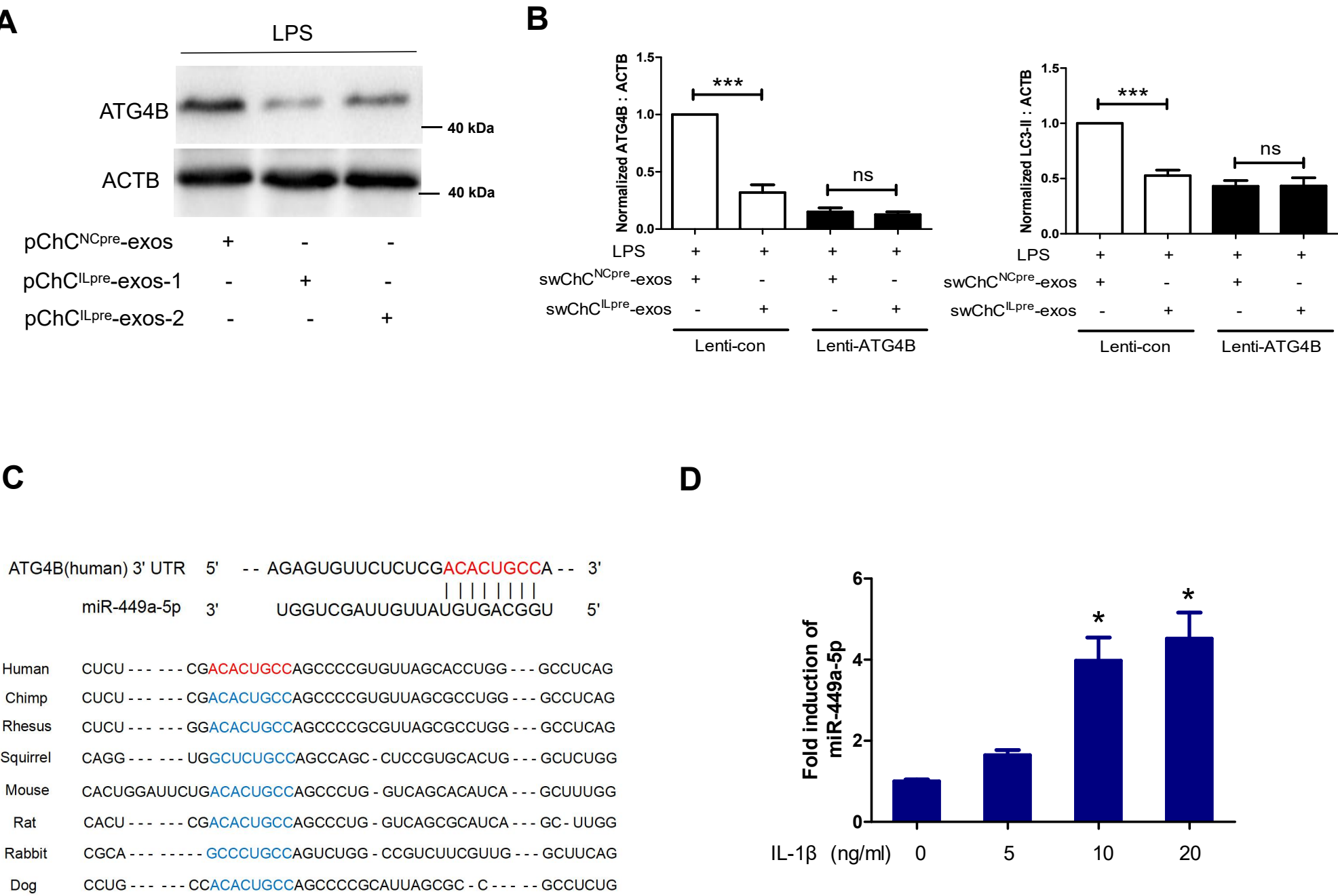

Supplement: Supplementary file 2 — Supplementary Figures [file 41419_2019_1739_MOESM2_ESM.pdf]
